# West-Nile virus encephalitis in an immunocompetent pediatric patient: successful recovery

**DOI:** 10.1186/s13052-018-0574-x

**Published:** 2018-11-20

**Authors:** Salvatore Savasta, Francesca Rovida, Thomas Foiadelli, Anna Maria Campana, Elena Percivalle, Gian Luigi Marseglia, Fausto Baldanti

**Affiliations:** 1 Pediatric Clinic, Fondazione IRCCS Policlinico San Matteo, V.le C. Golgi, 19 –, 27100 Pavia (PV), Italy; 2 Molecular Virology Unit, Microbiology and Virology Department, Fondazione IRCCS Policlinico San Matteo, 27100 Pavia, Italy; 3 Department of Clinical, Surgical, Diagnostic and Pediatric Sciences, University of Pavia, Pavia, Italy

**Keywords:** West Nile virus, Encephalitis, Pediatric, Flavivirus, Neuroinvasive disease, Italy

## Abstract

**Background:**

West Nile virus (WNV) is a mosquito-borne RNA virus belonging to the *Flaviviridae* family. Symptomatic infection happens in only about 20% of the cases, while WNV neuroinvasive disease (WNND) is rare and accounts for less than 1%. There is insufficient information about natural history and clinical course in children, because underdiagnosis is common, and reports are scarce. On the other hand, Europe has seen a dramatic increase of WNV infections in the last decades, and the Po valley itself, in Northern Italy, has become an endemic region since 2013.

**Case presentation:**

We hereby report a case of West-Nile virus neuroinvasive disease in a 12-year-old boy. This is one of the very few cases diagnosed in the Italian pediatric population. The clinical presentation was compatible with acute encephalitis. Diagnosis was made by detection of specific IgM in both serum and cerebrospinal fluid. He finally was discharged with complete recovery, and no neurologic sequelae after a 12-months follow up period.

**Conclusions:**

Given its non-specific clinical presentation, the diffusion of WNV constitutes a crucial and emerging concern. Even though rare, neuroinvasive WNV infection should always be suspected in pediatric patients, living or traveling in endemic areas, presenting with meningitis, encephalitis or acute flaccid paralysis during the WNV transmission season.

## Background

West Nile virus (WNV) is a mosquito-borne RNA virus belonging to the *Flaviviridae* family (genus *Flavivirus*) [1]. The main vector are mosquitoes belonging to the *Culex* genus which infects birds, the main WNV vertebrate hosts. WNV is maintained in nature due to mosquitoes and birds enzootic cycle, while humans and horses are accidental hosts [2]. The majority (~ 80%) of WNV infections in humans are asymptomatic. Symptomatic infections are mostly (~ 20%) associated with a mild, self-limiting febrile illness (WNF), whereas WNV neuroinvasive disease (WNND) develops in < 1% of WNV infected persons as meningitis, encephalitis or acute flaccid paralysis [3]. WNND mainly occurs in young adults and elderly patients. However, WNND have been also reported in children [3]. Natural history and clinical disease spectrum are not fully understood in pediatric patients due to a wide lack of reporting and underdiagnosis in children [3]. The highest epidemiological and clinical data on WNV infection in children have been described from the US, where large human outbreaks have occurred since 1999. On the contrary, less information is available from Europe, where human outbreaks have been limited to less cases of infection [3].

In this report, we describe a case of West-Nile virus encephalitis in a 12-years old immunocompetent child in the Lombardy Region, Northern Italy, a WNV endemic area since 2013 [4].

## Case presentation

In mid September 2014, a 12-year-old boy presented at the Pediatric Emergency Department of the Fondazione IRCCS Policlinico San Matteo, Pavia, due to persistent fever, headache and diffuse pruriginous erythematous rash. The child’s mother reported that a few days before the onset of symptoms he went fishing near the family country-house in the province of Pavia (Po valley - Lombardy Region). On preliminary examination, his vital signs and general medical examination were normal. A mildly altered mental status was noted. Neurologic examination was unremarkable except for photophobia, without other signs of meningitis. Biochemical tests showed hemoglobin 12.8 mg/dl, lactate dehydrogenase 251 mU/ml (reference range: 125–220 mU/ml) and C-reactive protein 0.79 mg/dl (reference range: 0.00–0.50 mg/dl). Meningoencephalitis was suspected. The patient was thus hospitalized in the Pediatric Department and empiric antiviral and antibacterial therapies with acyclovir and ceftriaxone were promptly started. Cerebrospinal fluid (CSF) analysis showed 180 cells, glucose of 63 mg/dl (reference range: 40–70 mg/dl) and protein of 63 mg/dl (reference range: 20–45 mg/dl). Bacterial blood and CSF cultures were negative. CSF was tested by real-time RT-PCR and PCR for the following neurotropic viruses: Herpes simplex, Enterovirus, Polyomavirus JC, Herpesvirus 6, WNV, Phleboviruses and Flaviviruses. Furthermore, serum and urine were analyzed with WNV real-time RT-PCR and Flavivirus RT-PCR. The molecular investigation of neurotropic virus genome was negative in all the biological samples. Phleboviruses and WNV-IgM and IgG antibodies detection was performed in both serum and CSF samples. WNV-IgM tested positive both in serum and CSF while WNV-IgG were negative: this was confirmative for an acute WNV infection. An electroencephalogram (EEG), performed within 24-h after admission, revealed encephalitic-like bilateral slow waves and brain magnetic resonance imaging (MRI) did not reveal any abnormality.

The patient’s conditions remained stable and after seven days of hospitalization he was discharged with complete recovery and EEG normalization. At discharge, WNV-IgM and WNV neutralizing antibodies were positive and a WNV-IgG seroconversion was observed. The child was followed-up for 12 months, to monitor the clinical and neurological evolution and evaluate the WNV antibodies kinetics. A complete recovery with no neurological sequelae was observed. Serum WNV-IgM persistence was still evident 12 months after the onset of the disease.

## Discussion and conclusions

The highest number of epidemiological and clinical data on WNV infection in pediatric patients have been conducted in the US, where large human outbreaks have occurred every year, after the first identification of the virus in 1999 [3]. Several survey studies [5, 6] of WNV infection in children (< 18 years of age), conducted in US in different periods from 1999 to 2012, demonstrated that the number of WNND in pediatric patients was about 3.8–4.0% of the total cases of symptomatic WNV infections. In Europe, less information is available about the prevalence and incidence of WNV infection in the pediatric population, probably due to the lower number of WNV human cases. In Italy, the first human cases of WNV infection have been reported in 2008. In the period 2008–2015, 173 indigenous cases of human WNND were diagnosed in Italy. Median age was 73 years (range: 10–90 years) during the entire surveillance period, varying from a minimum of 67 years (range: 41–68 years) in 2010 to 77 years (range: 42–89 years) in 2013 [7]. In the Veneto Region (North-Eastern Italy), during the period 2010–2014, 88 WNV infections were identified (46 WNND and 42 WNF). Only one patient aged < 18 years was reported (1.14%), with signs and symptoms of encephalitis [3]. Until 2013, Lombardy Region (Northern Italy) was marginally involved in WNV epidemics, when an outbreak of WNV infection occurred and eighteen cases were detected [4]. In the period 2013–2017, the Molecular Virology Unit of our hospital, as a Regional Reference Laboratory for the diagnosis of emerging viruses in the Lombardy Region, identified 72 indigenous WNV cases: the only reported pediatric patient is described in the present paper. It represents one of the very rare cases of WNND diagnosed in the pediatric population in Italy. The child came to our attention in September, when WNND tend to reach a seasonal peak in Italy [7]. Some days before the onset of symptoms the boy went fishing and during this activity he was exposed to mosquito bites in an area (Pavia province – Po valley) of the Lombardy Region which was affected by WNV-positive mosquitoes as reported by the WNV Surveillance [8]. We can hypothesize that WNV was transmitted to the child through the bite of infected mosquitoes, being this the most common way of transmission as well in adults as in children [7].

WNV infections are asymptomatic or subclinical in 70% of cases. In the pediatric population, symptomatic diseases presents mainly as WNF, while WNND occur in approximately 1% of all infections, presenting as meningitis, encephalitis or acute flaccid paralysis [2]. Compared with adults, clinical presentation of WNND in children is generally milder and followed by complete recovery (even if cases of permanent disability and deaths have been reported) [3]. Encephalitis has been more frequently reported in older age groups whereas meningitis is more commonly described in pediatric patients [9]. Moreover, detection of rash is unusual in patients with encephalitis [3], even though it has been reported as a prognostic indicator of severe disease and death [10]. The clinical presentation of our patient was compatible with encephalitis. Fortunately, the occurrence of a diffuse rash in our patient did not correlate with a negative outcome, as he was discharged with complete recovery after seven days of hospitalization.

In 2008, the European Union (EU) established the clinical and diagnostic criteria for probable and confirmed WNV case definition (European Centre for Disease Prevention and Control [ECDC]). A case is considered probable if the patient meets any of the following clinical criteria: suggestive clinical presentation (encephalitis, meningitis, fever) in the presence of WNV-specific immunoglobulin M (IgM) or IgG in serum with IgG seroconversion, or a four-fold increase in IgG titer on two subsequent samples. A confirmed case is defined as meeting the previous clinical criteria and one or more of the following additional criteria: (1) Isolation of WNV from blood or cerebrospinal fluid, (2) detection of WNV RNA in blood and/or CSF, (3) WNV-specific IgM in CSF, and (4) detection of WNV IgM at a high titer and detection of WNV IgG, confirmed by a neutralization assay. According to the EU criteria, our patient could be considered a confirmed case of WNND due to the detection of WNV IgM in CSF, collected in the symptomatic phase of the neurological infection and the subsequent WNV IgG seroconversion and positivity of WNV neutralizing antibodies.

As recommended by the CDC (Center of Disease Control and prevention), WNV therapy is mainly supportive because of the lack of a specific antiviral therapy [3]. Specifically, the patient’s treatment included infusion of fluid, antipyretic, antibiotic and antiviral drugs. Remarkably, our patient successfully recovered after the acute phase and did not present any neurological sequelae during the follow-up.

In conclusion, WNV is responsible of an increasing number of neuro-invasive infections all over the world. Given its non-specific clinical presentation, the diffusion of WNV constitutes a crucial and emerging concern. Our case underlines that, even though WNND is rare, WNV-infection should be always suspected in pediatric patients, living or traveling in endemic areas, presenting with meningitis, encephalitis or acute flaccid paralysis during the WNV transmission season. Nevertheless, more detailed analysis and reports of WNND pediatric cases are needed to clarify clinical aspects and increase knowledge and awareness over this emergent disease among physicians.

## Abbreviations

CDC: Center of Disease Control And Prevention
CSF: Cerebrospinal Fluid
EEG: Electroencephalogram
WNF: West Nile Febrile Illness
WNND: West Nile Virus Neuroinvasive Disease
WNV: West Nile Virus
